# Utilization Patterns of Nebulized Glycopyrronium in Patients Hospitalized for Acute Exacerbations of Obstructive Airway Disease (AEOAD)—Indian Expert Perspectives

**DOI:** 10.3390/arm94040043

**Published:** 2026-06-29

**Authors:** Arjun Khanna, Pradyut Waghray, Ashok Kr Singh, Jinay Mehta, Rithik, Sagar Bhagat, Saiprasad Patil, Hanmant Barkate

**Affiliations:** 1 Department of Pulmonary Medicine, School of Medicine, Amrita Vishwa Vidyapeetham, Faridabad 121002, Haryana, India; 2 Pulmonary Medicine SVS Medical College, Mahbubnagar 509001, Telangana, India; 3 Kunal Institute of Medical Specialties Pvt Ltd., Hyderabad 500004, Telangana, India; 4 SR., Pulmonology Apollo Hospitals Hyderabad, Hyderabad 500033, Telangana, India; 5 Pulmonary & Critical Care Medicine Regency Hospital, Kanpur 208005, Uttar Pradesh, India; 6 Global Medical Affairs, Glenmark Pharmaceuticals, Ltd., Mumbai 400099, Maharashtra, India

**Keywords:** acute exacerbation, obstructive airway disease, nebulized glycopyrronium, long-acting muscarinic antagonist, triple therapy, consensus statement, inpatient management, India-specific evidence

## Abstract

**Highlights:**

**What are the main findings?**

- Early use of nebulized glycopyrronium as part of triple therapy (with formoterol and budesonide) provides rapid, sustained bronchodilation with a favourable cardiovascular profile, and is preferred after initial stabilization with short-acting bronchodilators.
- Its use is associated with improved clinical and operational outcomes, including reduced hospital stay and readmissions, decreased reliance on rescue medications and steroids, and lower nursing workload with improved patient satisfaction.

**What are the implications of the main findings?**

- Nebulized glycopyrronium may serve as a well-tolerated and more effective alternative to frequent short-acting bronchodilator use in hospitalized AEOAD patients.
- Early incorporation into treatment pathways can optimize inpatient care, accelerate recovery, and streamline discharge planning.

**Abstract:**

**Background:** Acute exacerbation of obstructive airway disease (AEOAD) is a major cause of hospitalization, morbidity, and premature mortality in India. Hospitalized patients for the same are predominantly treated with short-acting bronchodilators, which require frequent administration and are associated with systemic adverse effects. Despite the availability of nebulized long-acting muscarinic antagonists (LAMAs) with quick onset of action, such as glycopyrronium, their role in acute care remains unclear in India. **Methods:** A pan-India expert opinion-building initiative was conducted among 220 pulmonologists across Tier I–II cities through 13 structured advisory meetings between April 2025 and July 2025. The final expert perspectives were then categorized into recurrent insights, raised in 75% or more meetings, and variable insights, raised in <75% of all meetings. **Results**: Experts reported that AEOAD management commonly involved initial stabilization with SABA/SAMA followed by transition to triple therapy with nebulized glycopyrronium, formoterol, and budesonide. Nebulized glycopyrronium was perceived to provide rapid and sustained bronchodilation with fewer cardiovascular side effects compared to short-acting agents. Benefits were reported in patients with frequent exacerbations, high sputum burden, and bronchiectasis. Operational advantages included reduced dosing frequency and nursing workload. Experts also noted potential improvements in hospital stay and readmissions; however, these observations were based on clinical experience rather than controlled data. **Conclusions:** Indian pulmonologists agreed that early initiation of nebulized glycopyrronium (with formoterol and budesonide) in hospitalized AEOAD may improve symptom control, lower exacerbation burden, reduce reliance on short-acting bronchodilators and corticosteroids, and shorten hospital stays.

## 1. Introduction

Asthma and chronic obstructive pulmonary disease (COPD), collectively referred to as obstructive airway diseases (OADs), remain one of the major public health concerns in India, contributing substantially to morbidity, healthcare utilization, and economic burden [1,2,3]. Acute exacerbations of OADs are the key drivers of disease progression, often requiring hospitalization. Optimizing the use of bronchodilators during these episodes is critical to improve clinical outcomes and reduce healthcare burden. Short-acting bronchodilators, including short-acting β2-agonists (SABAs) and short-acting muscarinic antagonists (SAMAs), form the cornerstone of initial management of acute exacerbations due to their rapid onset of action. However, their short duration of action necessitates frequent administration. This increases nursing workload and overall treatment burden. Repeated high-dose use may also predispose patients to systemic adverse effects such as tremors, hypokalemia, and arrhythmias [4,5,6,7,8,9].

In contrast, long-acting muscarinic antagonists (LAMAs) provide sustained bronchodilation lasting 12–14 h by selectively inhibiting M3 receptors in airway smooth muscles. Current international guidelines [10] recommend the use of short-acting bronchodilators for immediate symptom relief, followed by escalation to long-acting agents as maintenance therapy after initial stabilization.

Glycopyrronium, a LAMA, possesses a relatively rapid onset of bronchodilatory action in addition to prolonged duration, making it potentially suitable for earlier introduction even in acute care settings. It is highly selective for the M3 subtype, resulting in effective bronchodilation with a reduced propensity for cardiovascular adverse effects [11].

From a practical standpoint, the use of nebulized LAMA therapy in the inpatient setting may offer advantages such as reduced dosing frequency, improved bronchodilator coverage over 24 h, and potential reduction in reliance on frequent SABA/SAMA administration. However, in real-world settings, the optimal timing of LAMA initiation during hospitalization and its potential role earlier in the treatment continuum remain less clearly defined.

Despite these potential advantages, the absence of structured hospital protocols for initiating nebulized LAMA therapy during acute exacerbations has led to variability in clinical practice across institutions. Therefore, this expert opinion paper aimed to assess current utilization patterns, clinical experiences, and expert perspectives on nebulized glycopyrronium in hospitalized patients with AEOAD across the inpatient continuum, including emergency care, ward management, discharge, and post-discharge settings.

## 2. Methodology

From April 2025 to July 2025, 13 virtual advisory meetings (focus group discussions, FGDs) were conducted with 220 panellists, primarily practicing pulmonologists, representing northern, southern, western, eastern, and central regions of India, to explore utilization patterns, clinical perspectives, and real-world experiences with nebulized glycopyrronium in patients hospitalized for acute exacerbations of obstructive airway disease (AEOAD).

Panellists were invited by email. Inclusion criteria required practicing pulmonologists with ≥10 years’ clinical experience who actively managed hospitalized AEOAD patients. Participants represented a range of practice settings across Tier I–II cities, including Mumbai, Delhi, Bengaluru, Chandigarh, Patna, Lucknow, Kolkata, Jaipur, Hyderabad, Chennai, Ernakulam, Ahmedabad, and Pune.

Discussions were structured around six clinical practice domains:

- Clinical practice and experience;
- Patient profile;
- Comparative evaluation with other bronchodilators;
- Operational feasibility;
- Outcomes and monitoring in real-world settings;
- Current evidence and knowledge gaps.

**Figure 1:**
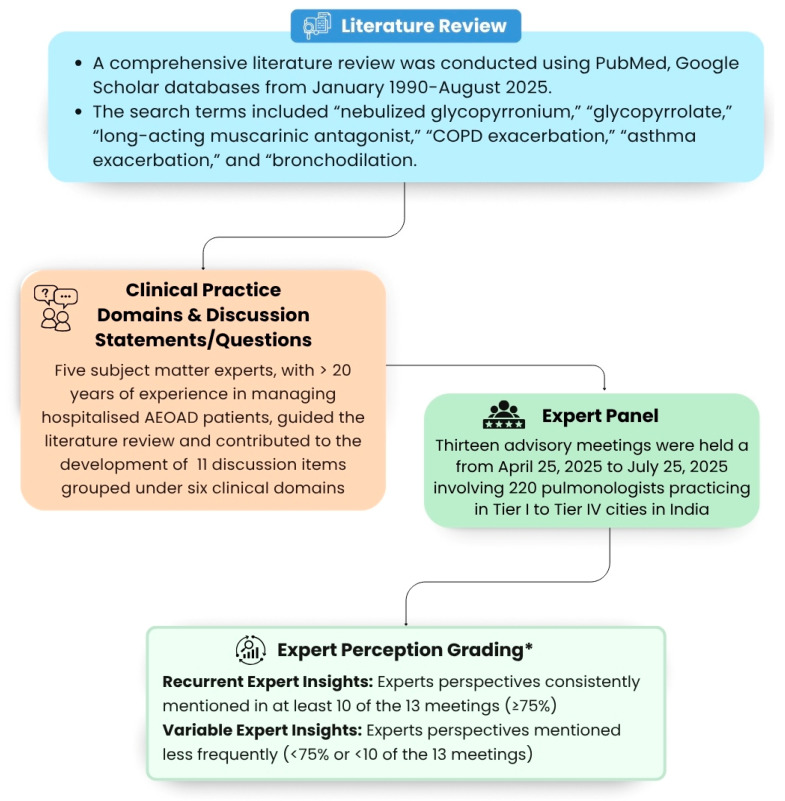
**Study methodology flow diagram.** A schematic representation of the literature review, expert panel discussions, and expert perception grading process used in this study.

A panel of five subject matter experts (each with >20 years’ experience managing hospitalized AEOAD patients) reviewed the literature and developed 11 discussion items grouped into the six domains. The experts validated the questionnaire items for clarity and relevance to assess prescribing practices and integration of nebulized glycopyrronium into hospital care pathways (see Figure 1). The clinical practice domains and corresponding discussion statements are presented in Table 1.

A comprehensive literature search of PubMed and MEDLINE (January 1990–August 2025) was performed using terms including “nebulized glycopyrronium,” “glycopyrrolate,” “long-acting muscarinic antagonist,” “COPD exacerbation,” “asthma exacerbation,” “hospitalized patients,” and “bronchodilation.” The review was supplemented by the Global Initiative for Chronic Obstructive Lung Disease (GOLD) 2026 guidelines and landmark trials such as the GOLDEN studies.

As the project collected expert opinion rather than primary patient data, institutional ethics committee approval was deemed unnecessary in accordance with the Indian Council of Medical Research National Ethical Guidelines for Biomedical and Health Research Involving Human Participants (2017).

Insights from the 13 advisory meetings (no of KOLs and which specialties) were collected through guided discussions rather than formal voting or live polling. All sessions were digitally recorded; recordings were transcribed and reviewed in detail. Each transcript was listened to at least twice by two independent reviewers to ensure transcription accuracy and reviewer familiarity with the data. Final expert perspectives were categorized into recurrent and variable insights:

- Recurrent Expert Insights: An insight was classified as recurrent when ≥75% of experts across ≥10 of the 13 meetings shared similar perspectives on a discussion point. Although derived from open discussion rather than formal scoring, the 75% threshold aligns with commonly used consensus methodologies [12,13].
- Variable Expert Insights: An insight was classified as variable when <75% of experts in <10 of the 13 meetings expressed differing perspectives on a discussion point, reflecting diversity in clinical experience, regional practice patterns, or patient profiles [14,15].

## 3. Results

The expert panel discussions featured balanced participation from the North, South, East, West, and Central regions of India, covering Tier I–II cities. This diversity reflects real-world practice and the potential benefits of nebulized glycopyrronium in hospitalized patients with AEOAD. Table 2 provides a comprehensive overview of expert perspectives on the early use of nebulized glycopyrronium in hospitalized patients with AEOAD, including the clinical rationale, patient selection criteria, and other operational and therapeutic factors.

The potential advantages of initiating early nebulized glycopyrronium include rapid bronchodilation, which may reduce the severity of AEOAD and support a smooth transition to maintenance therapy (Table 3).

## 4. Discussion

This nationwide expert panel captured Indian pulmonologists’ perspectives on nebulized glycopyrronium in hospitalized AEOAD, providing context-specific insights that complement global evidence. The discussions identified clinical, operational, and patient-related factors influencing prescribing practices and highlighted key evidence gaps and the need for locally relevant protocols. Panel perspectives across six domains were compared with existing literature to inform future research and guideline development.

### 4.1. Clinical and Practical Experience

Most panelists perceived that for initial stabilization in patients admitted due to acute exacerbations, SABA/SAMA is preferred, followed by the use of nebulized glycopyrronium along with formoterol and budesonide once the patient stabilizes. This approach aligns with the GOLD 2026 guidelines for management of COPD recommendations, which emphasize the use of short-acting bronchodilators for immediate symptom relief during exacerbations and long-acting bronchodilators for maintenance therapy once the patient is stabilized [11].

A physician survey by Bhojwani et al. mirrored these findings: most clinicians preferred nebulized SABA + SAMA in the ER/ICU, and after stabilization, 44% favored nebulized glycopyrronium-based open triple therapy (with formoterol and budesonide) while 26% preferred glycopyrronium-based triple therapy via pMDI/DPI [14].

Regarding the use of short-acting bronchodilators, it was opined by the pulmonologist that repeated use of short-acting bronchodilators (SABA/SAMA) was sometimes associated with hypokalemia and tachycardia, which was concerning because cardiac-related complications, including atrial fibrillation, remain the leading cause of mortality in COPD patients [15].

Similar results were found in a systematic review done by Kopsaftis et al., which suggested that no additional benefits were observed while using higher doses of short-acting β2-agonists, but there was a small increase in adverse events for participants using higher doses of β2-agonists used in patients [9]. In contrast, the panelists highlighted that nebulized glycopyrronium demonstrated reduced cardiovascular adverse effects when used for patients hospitalized for acute exacerbations. This attribute of glycopyrronium is due to its high selectivity for M3 receptors, ensuring effective airway relaxation while minimizing interaction with M2 receptors, thereby reducing cardiovascular risks [11].

Nebulized glycopyrronium provides effective bronchodilation with a more favourable cardiovascular safety profile than repeated high-dose short-acting bronchodilators, making it a safer option for hospitalized patients.

### 4.2. Patient Profile

It was widely opined that nebulized glycopyrronium was widely used in patients with frequent exacerbations of OAD, hospitalized for AEOAD, and with asthma-COPD overlap syndrome (ACOS). Similar results were observed in the drug utilization study conducted by Korukonda et al., which concluded that glycopyrronium, when added to LABA/ICS and used for incremental bronchodilation, showed significant improvement in FEV1 in patients with severe COPD and ACOS [16]. And in a study conducted by Panigrahi et al. and Patel et al., which reported that in India, glycopyrronium along with formoterol and budesonide containing triple nebulization was the preferred treatment for hospitalized patients with AEOAD, and it leads to clinically significant improvements in FEV_1_ without evident side effects [17,18].

The sustained bronchodilation further contributes to improved airflow, decreased hyperinflation, and enhanced patient comfort, which was also observed in the study conducted by Nardini et al. [19]. It was also opined that patients who were already on glycopyrronium prior to hospitalization should continue the same for better symptom control, especially those with bronchiectasis or high sputum burden, as LAMAs help reduce mucus plugging and improve airway clearance.

In regard to ventilated patients, nebulized glycopyrronium has been reported to reduce secretions; similar findings were found in a study by Priya et al. that showed glycopyrronium reduces airway secretions and improves ventilator compliance, aiding in weaning and reducing ICU burden [20].

For home nebulization in patients with obstructive airway disease, the pulmonologists had a collective opinion that nebulized glycopyrronium along with formoterol and budesonide is preferable for patients with cognitive impairments, neuromuscular disorders, arthritis, tremors, or Parkinson’s disease, as these individuals often struggle with handheld inhalers. This insight is supported by expert consensus conducted by Talwar et al., which highlights the operational and clinical benefits of nebulized long-acting bronchodilators in patients with physical or cognitive limitations [21]. These findings highlight that nebulization with glycopyrronium offers both clinical and practical advantages, supporting its broader role in improving AEOAD care in inpatient, outpatient, and home settings after discharge from the hospital.

### 4.3. Comparative Evaluation

The study conducted in critically ill mechanically ventilated patients of chronic obstructive pulmonary disease by Priya et al. reported that glycopyrronium nebulization resulted in fewer respiratory secretions, a longer duration of action (10–12 h) compared to salbutamol/ipratropium combinations, which typically last only 4–6 h, and is also characterized by reduced airway resistance and no adverse effects, such as hypertension, tachycardia, or dehydration, compared to salbutamol and ipratropium [20].

These findings further validate the panel perceptions in the current study, suggesting that nebulized glycopyrronium not only provides a rapid onset of action and more sustained bronchodilation but also demonstrates a better safety profile compared with other SABAs/SAMAs and LAMAs.

### 4.4. Operational Considerations

Unlike short-acting bronchodilators that require dosing every 4–6 h, glycopyrronium can be administered twice daily. This reduction in administration frequency decreases the time nurses spend on medication preparation, delivery, and cleaning of nebulizer equipment [22].

Reduced nebulization frequency also contributes to improved patient satisfaction and adherence. Patients often report greater improvement with health-related quality of life (HRQoL) scores when treatment schedules are simplified. Similar improvement in HRQoL scores was also observed in GOLDEN studies [23].

Finally, decreasing the frequency of nebulization leads to better infection control in shared ward environments. Frequent nebulization can increase aerosol generation, potentially leading to droplet and airborne transmission of pathogens. By minimizing nebulization frequency, glycopyrronium indirectly lowers cumulative exposure risk for healthcare workers and other patients [24].

### 4.5. Outcomes and Monitoring

The panelists had a collective opinion that switching to nebulized therapy with glycopyrronium in patients hospitalized for acute exacerbations may reduce hospital stays, use of rescue inhalers, readmissions, corticosteroid use, and ICU transfers, while also improving QoL. Similar results were seen in a study published by Khanna et al., which showed that when nebulized glycopyrronium was used, there was decreased reliance on SABA rescue inhalers, and the average ICU stay and ward stay of subjects was also shorter [25] compared to when salbutamol and ipratropium were used [26,27].

### 4.6. Evidence Required

Although the SABA/SAMA combination is traditionally preferred for AEOAD, early initiation with glycopyrronium/formoterol/budesonide triple nebulization is recommended by the panelists in the treatment of acute exacerbations of OAD. The panelists agreed that while clinical impressions are promising, India-specific randomized clinical trials (RCTs) and registries are necessary to validate the efficacy, cost-effectiveness, and optimal dosing strategies of nebulized glycopyrronium.

#### 4.6.1. Additional Considerations

In neurology ICUs, nebulized glycopyrronium improves ventilator compliance and reduces suctioning frequency. Its use has also been extended beyond pulmonology, with cardiologists and general physicians adopting it for high-burden cases. As nebulized glycopyrronium is non-invasive and free of central adverse effects, it does not cross the blood–brain barrier, unlike other anticholinergics. One case report demonstrated its effectiveness in reducing drooling, providing symptomatic relief without significant adverse effects [28].

Two cases of posterior drooling treatment were reported (an 82-year-old male stroke patient and a 1-year-old female with cerebral palsy), and salivary aspiration and the effect of nebulized glycopyrronium were identified using radionuclide sialograms. Treatment with nebulized glycopyrronium was well tolerated, with reduced posterior drooling, improved airway safety, and reduced aspiration risk [29].

#### 4.6.2. Potential Implications for Indian Practice

**Figure 2:**
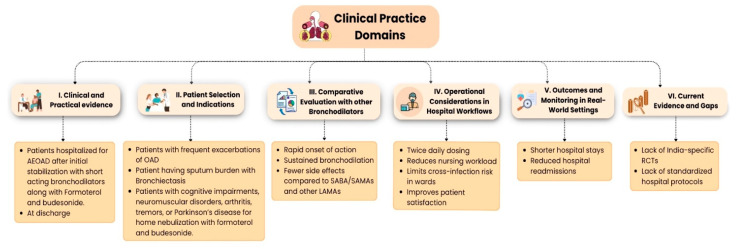
Clinical considerations for nebulized glycopyrronium in hospitalized patients with AEOAD—a conceptual framework. Abbreviations: AEOAD—acute exacerbation of obstructive pulmonary disease, FEV1—forced expiratory volume in one second, ER—emergency room, ICU—intensive care unit, LABA/LAMA—long-acting beta-2 agonists/long-acting muscarinic antagonists.

Given the variability in hospital infrastructure, prescription patterns, and patient profiles across India, understanding the role of nebulized glycopyrronium is key to its rational use in clinical practice. The proposed conceptual framework (Figure 2) was developed from an expert perspective. By providing consistent bronchodilation regardless of patient effort, nebulized glycopyrronium delivers a reliable dose during AEOAD care, facilitating rapid stabilization and supporting timely discharge. This framework may guide the real-world practices of Indian pulmonologists and intensivists, thereby improving AEOAD care (Figure 2).

#### 4.6.3. Strengths and Limitations

The first nationwide expert opinion was captured through the perspectives of 220 pulmonologists at 13 structured meetings covering diverse regions and practice settings. Structured domains (clinical practice, patient selection, comparative evaluation, operational issues, outcomes, and evidence gaps) ensured comprehensive thematic analysis. The expert-validated discussion framework improved the consistency and credibility of these insights. However, this has some limitations. It represents expert perspectives rather than randomized clinical trial data, limiting its generalizability. Another limitation was the absence of a direct comparison group and quantitative outcome measures, and the findings were based solely on the panelists’ perceptions and clinical experiences. A key limitation is the absence of cost-effectiveness data; in resource-constrained settings such as India, cost and availability are important determinants of adoption, and no formal economic evaluation was performed. To support our findings, we recommend conducting a multicenter study to optimize the use of nebulized glycopyrronium in hospitalized patients with AEOAD in India. Longitudinal studies should be performed to evaluate the efficacy of nebulized glycopyrronium, focusing on its impact on hospital stay and readmission compared with SABA/SAMAs and other LAMA regimens. It is also crucial to evaluate nebulized glycopyrronium dosing strategies in both inpatient and outpatient settings and to conduct cost-effectiveness studies comparing them with SABA/SAMAs and other LAMA regimens.

## 5. Conclusions

This expert consensus highlights the potential of nebulized glycopyrronium in addressing critical gaps in the management of patients hospitalized with AEOAD in India. The panelists agreed that early initiation provides sustained bronchodilation, reduces reliance on short-acting agents, and offers operational benefits, such as a lower nursing workload and smoother discharge transitions. However, these expert perspectives, rather than formal recommendations, should be interpreted with caution.

## Figures and Tables

**Table 1:** Clinical Practice domains and discussion Questions/Statements.

Clinical Practice Domains	Questions/Statements
**1. Clinical Practice and Experience**	1. Have you used nebulized glycopyrronium in hospitalized patients with AE-OAD? If yes, in what clinical scenarios? When Immediately on arrival in ER/ICU After stablization in ER/ICU Once shifted in ward At discharge How Monotherapy or in combination 2. How does your current hospital protocol manage patients with AE-OAD in terms of anticholinergic therapy? 3. What is your experience with the onset of action, symptom control, and patient response with nebulized glycopyrronium?
**2. Patient profile**	4. What patient profiles would benefit most from nebulized glycopyrronium during acute exacerbation? 5. Do you consider it more useful in acute exacerbation of COPD, asthma, or both? Why? 6. Are there any specific contraindications or concerns you have observed with its use in hospitalized patients?
**3. Comparative Evaluation**	7. How does glycopyrronium compare to other LAMAs in efficacy and safety in the acute setting? 8. Have you used glycopyrronium in combination with SABA/SAMA or ICS/LABA?
**4. Operational considerations**	9. How does the use of nebulized glycopyrronium impact nursing workload, administration time, or patient satisfaction?
**5. Outcomes & Monitoring**	10. Have you observed any impact on hospital length of stay, readmission rates, or need for ICU transfer with its use?
**6. Evidence**	11. What additional evidence or data would you need to support broader adoption of nebulized glycopyrronium in hospital settings?

Abbreviations: AEOAD—acute exacerbations of obstructive airway disease; ER—emergency room; ICU—intensive care unit; COPD—chronic obstructive pulmonary disease; SABA/SAMA—short-acting beta-two agonists/short-acting muscarinic antagonists; LABA/LAMA—long-acting beta-two agonists/long-acting muscarinic antagonists.

**Table 2:** Summary of expert perspectives on early initiation of nebulized glycopyrronium in hospitalized patients with AEOAD.

Clinical Domains	Expert Perspectives
**Clinical Practice and Experience**	**Recurring Expert Insights:** Initial stabilization of AEOAD is typically achieved using SABA/SAMA nebulization. Following stabilization, patients are commonly transitioned to a triple therapy regimen (glycopyrronium, formoterol, and budesonide) during ward management and at discharge. Nebulized glycopyrronium provides rapid onset with sustained bronchodilation and is frequently used in combination with LABA and ICS. Frequent use of SABA/SAMA may be associated with adverse effects such as tachycardia and hypokalemia. The primary cause of mortality in COPD patients is cardiac origin, often with atrial fibrillation. Nebulized glycopyrronium combined with formoterol and budesonide did not show cardiovascular side effects, leading to reduced mortality in AEOAD patients. Lack of standardized hospital protocols for anticholinergic use leads to variability in prescribing practices. Glycopyrronium may be preferred in selected patients, particularly those with cardiovascular comorbidities, based on its favorable safety profile. **Variable Expert Insights** Nebulized therapy at discharge is guided by peak inspiratory flow and patient ability to use inhaler devices (MDI/DPI). Glycopyrronium may be considered in patients with prior LAMA exposure or inadequate response to short acting bronchodilators. In cases of diagnostic uncertainty, short-acting bronchodilators may be preferred.
**Patient profile**	**Recurring Expert Insights:** Nebulized glycopyrronium is considered useful in acute exacerbations of COPD, asthma, and asthma–COPD overlap. Patients previously on glycopyrronium may continue therapy, particularly those with bronchiectasis or high sputum burden, often in combination with LABA and ICS. Contraindications include hypersensitivity, narrow-angle glaucoma, paralytic ileus, and urinary retention. Caution is advised in patients with myasthenia gravis and during pregnancy. Benefits are seen across all patient groups, including mild to severe exacerbations due to its fast acting mechanism. It may also be beneficial in elderly patients with low peak inspiratory flow rate. **Variable Expert Insights** Use of LAMAs, including nebulized glycopyrronium, has extended to broader clinical settings, including patients with high sputum burden and complex comorbidities. In cases of contraindications, short-acting agents such as ipratropium may be preferred. Nebulized glycopyrronium may help reduce airway secretions in ventilated patients. It may be preferred in patients with cognitive impairment neuromuscular disorders, arthritis, tremors, or Parkinson’s disease post discharge from hospital for home nebulization as in such situation difficulty is seen while using inhalers.
**Comparative Evaluation**	**Recurring Expert Insights:** Glycopyrronium is perceived to offer effective bronchodilation with a favorable safety profile compared to short-acting bronchodilators and other LAMA/LABA therapies. It demonstrates a relatively rapid onset of action along with sustained bronchodilation. High M3 receptor selectivity may contribute to fewer cardiovascular adverse effects such as tachycardia and palpitations. **Variable Expert Insights** Critical care consultants are concerned about the effects of glycopyrronium on dryness and mucus plugs in intubated patients. Individualized treatment decisions should consider the underlying cause of exacerbation and the need for adjunct therapies such as inhaled corticosteroids. Clinical judgment, including regular assessment and auscultation, is important to guide the therapy and ensure appropriate tapering of bronchodilators.
**Operational Considerations**	**Recurring Expert Insights:** Nebulized glycopyrronium enables reduced dosing frequency (typically twice daily), which may decrease nursing workload and administration burden. Glycopyrronium has been shown to enhance the quality of life of patients. The rapid onset of action within three to five minutes and increased duration of action lead to reduced nebulization frequency and improved patient satisfaction. The reduced frequency of nebulization also potentially lowers the risk of droplet transmission in shared ward environments, thereby enhancing infection control measures. **Variable Expert Insights** Glycopyrronium reduces secretions and suctioning in ventilated patients.
**Outcome & Monitoring**	**Recurring Expert Insights:** Transition to nebulized glycopyrronium in combination with formoterol and budesonide may be associated with improvements in clinical outcomes such as reduced hospital stay, readmissions, and need for intensive care interventions. These observations are primarily based on expert clinical experience rather than controlled evidence. **Variable Expert Insights** Continued use of glycopyrronium post-discharge is associated with reduced exacerbations and related hospitalizations. Patients who were prescribed home glycopyrronium nebulization upon discharge showed improvement, with reduced future exacerbations and exacerbation related hospitalization. Administering the medication once daily may not be sufficiently effective for patients; therefore, a twice-daily regimen is likely to be more beneficial.
**Evidence**	Multicentric randomized controlled trials/real-world studies are necessary to validate the regular use of nebulized glycopyrronium in AEOAD patients with a varied demographic profile.
**Additional Considerations**	Neuro patients such as those with stroke, Parkinson’s disease, or neuromuscular weaknes soften experience microaspirations that lead to increased airway secretions, making ventilation more difficult; nebulized glycopyrronium helps by reducing these secretions, improving ventilator compliance, and supporting faster weaning.

Abbreviations: AEOAD—acute exacerbations of obstructive airway disease; ER—emergency room; ICU—intensive care unit; COPD—chronic obstructive pulmonary disease; SABA/SAMA—short-acting beta-two agonists/short-acting muscarinic antagonists; LABA/LAMA—long-acting beta-two agonists/long-acting muscarinic antagonists; ICS—inhaled corticosteroids; OD—once daily; BD—twice daily.

**Table 3:** Summary of expert perspectives on the potential advantages of early initiation of nebulized glycopyrronium in hospitalized patients with AEOAD.

Potential Advantages of Early Initiation of Nebulized Glycopyrronium
Rapid bronchodilator effects enable rapid symptom relief. Prevents the worsening of acute airflow limitation, reducing the severity of exacerbations. Due to the long duration of action, the twice daily (BD) dosing burden on nursing staff is reduced, a practical advantage in a hospital setting. Demonstrates good safety and tolerability with fewer adverse effects. Supports a smooth transition to maintenance therapy by providing sustained bronchodilation.

Abbreviations: AEOAD—acute exacerbations of obstructive airway disease, BD—twice daily, SABA/SAMA—short-acting beta-two agonists/short-acting muscarinic antagonists.

## Data Availability

The data presented in this study are available on request from the corresponding author due to privacy and confidentiality restrictions associated with expert opinion discussions.
